# Author's Reply

**DOI:** 10.1371/journal.pmed.0020398

**Published:** 2005-11-29

**Authors:** John P. A Ioannidis

**Affiliations:** **1**University of Ioannina School of MedicineIoanninaGreece

I agree with Ian Shrier [1] that, when the effect size is large, adequate power is obtained with small numbers, and it is unnecessary to aim at very large studies. However, most effect sizes probed with statistical testing seem to be small. I also agree that heterogeneity is useful and can offer valuable insights [2]. Sometimes heterogeneity can show us that there are actually two or more research questions, where we thought there was only one [3]. The danger is when heterogeneity is silenced and dismissed in favor of claiming consistent results and when heterogeneity is exploited to show only the most spectacular results—unfortunately, this is not uncommon.

As Stephen Pauker [4] also points out correctly, it is useful to think about what the post-study odds are that one is aiming for if a study eventually were to get a “positive” result. Some residual uncertainty is unavoidable in any research question, no matter how strong the evidence. We should learn to live with uncertainty. I also agree that often the credibility level is less than 50%, yet decisions still have to be made. I don't see a problem implementing a very safe and very cheap medical intervention, even if the credibility that it is effective is only 20%. However, it is important to understand and acknowledge that this intervention has a credibility of 20%, while another has a credibility of 70%. I have no objection or preference on how exactly this will be calculated and plotted. Likelihood ratios are also a nice equivalent approach to calculate the probabilities or odds.

I agree with Jonathan D. Wren [5] that it is impossible to be 100% certain about the exact pre-study odds of truth for any research, mine included of course. However, I argue that we need to start thinking more seriously and consistently about these pre-study odds. In the single nucleotide polymorphism (SNP) association example, one might argue that 1:10,000 is not the best choice, but I doubt anyone would choose 1:100 [6]. Some fields may, indeed, have a pre-study odds of zero—these are the “null fields” that I discussed [7]. The differences in the range in pre-study odds are huge in current research, and I am afraid that this is almost completely ignored. I also have no objection about the framework concept. It is nice to see multiple lines of evidence converge. In fact “framework” evidence may be used to formulate more accurate pre-study odds. However, we should be cautious about how this framework is interpreted. We need more empirical data on how scientists try to converge various pieces of biological, epidemiological, and clinical information. I suspect that bias to make things fit, even if they don't, is not negligible.
